# Beyond Maastricht IV: are standard empiric triple therapies for *Helicobacter pylori* still useful in a South-European country?

**DOI:** 10.1186/s12876-015-0245-y

**Published:** 2015-02-15

**Authors:** Nuno Almeida, Maria Manuel Donato, José Manuel Romãozinho, Cristina Luxo, Olga Cardoso, Maria Augusta Cipriano, Carol Marinho, Alexandra Fernandes, Carlos Calhau, Carlos Sofia

**Affiliations:** 1 Gastroenterology Department, Centro Hospitalar e Universitário de Coimbra, Praceta Mota Pinto e Avenida Bissaya Barreto, 3000-075 Coimbra, Portugal; 2 Gastroenterology Centre, Faculty of Medicine, Coimbra University, Praceta Mota Pinto e Avenida Bissaya Barreto, 3000-075 Coimbra, Portugal; 3 Laboratory of Microbiology, Faculty of Pharmacy, Coimbra University, Azinhaga de Santa Comba, 3000-548 Coimbra, Portugal; 4 Pathology Department, Coimbra University Hospital Centre, Praceta Mota Pinto e Avenida Bissaya Barreto, 3000-075 Coimbra, Portugal

**Keywords:** Clarithromycin, Compliance, *Helicobacter pylori*, Levofloxacin, Treatment failure

## Abstract

**Background:**

Empiric triple treatments for *Helicobacter pylori* (*H. pylori*) are increasingly unsuccessful. We evaluated factors associated with failure of these treatments in the central region of Portugal.

**Methods:**

This single-center, prospective study included 154 patients with positive ^13^C-urea breath test (UBT). Patients with no previous *H. pylori* treatments (Group A, n = 103) received pantoprazole 40 mg 2×/day, amoxicillin 1000 mg 12/12 h and clarithromycin (CLARI) 500 mg 12/12 h, for 14 days. Patients with previous failed treatments (Group B, n = 51) and no history of levofloxacin (LVX) consumption were prescribed pantoprazole 40 mg 2×/day, amoxicillin 1000 mg 12/12 h and LVX 250 mg 12/12 h, for 10 days. *H. pylori* eradication was assessed by UBT 6–10 weeks after treatment. Compliance and adverse events were assessed by verbal and written questionnaires. Risk factors for eradication failure were determined by multivariate analysis.

**Results:**

Intention-to-treat and per-protocol eradication rates were Group A: 68.9% (95% CI: 59.4–77.1%) and 68.8% (95% CI: 58.9–77.2%); Group B: 52.9% (95% CI: 39.5–66%) and 55.1% (95% CI: 41.3–68.2%), with 43.7% of Group A and 31.4% of Group B reporting adverse events. Main risk factors for failure were *H. pylori* resistance to CLARI and LVX in Groups A and B, respectively. Another independent risk factor in Group A was history of frequent infections (OR = 4.24; 95% CI 1.04–17.24). For patients with no *H. pylori* resistance to CLARI, a history of frequent infections (OR = 4.76; 95% CI 1.24–18.27) and active tobacco consumption (OR = 5.25; 95% CI 1.22–22.69) were also associated with eradication failure.

**Conclusions:**

Empiric first and second-line triple treatments have unacceptable eradication rates in the central region of Portugal and cannot be used, according to Maastricht recommendations. Even for cases with no *H. pylori* resistance to the used antibiotics, results were unacceptable and, at least for CLARI, are influenced by history of frequent infections and tobacco consumption.

## Background

Approximately 50% of the world population is infected by *Helicobacter pylori* (*H. pylori*). This bacterium is responsible for multiple gastric diseases, including gastroduodenal ulcer, gastric adenocarcinoma and mucosa-associated lymphoid tissue lymphoma [1-3]. After almost thirty years of treating *H. pylori*, the ideal regimen has not been found. Some consensus conferences have recommended treatments with cure rates of ≥80% on an intention-to-treat (ITT) basis [4]. More recently, regimens of at least 90–95% efficacy, and preferably 95–100%, have been suggested as the standard of care [5].

Although meta-analyses have reported a decreased effectiveness for triple regimens containing clarithromycin (CLARI), the so-called “legacy triple therapy” is still widely used in several countries; the last Maastricht consensus maintained it as a first-line treatment under certain conditions [2,5,6]. The main reason for failure of this regimen is increased resistance to CLARI [2,7]. In Europe, primary *H. pylori* resistance to this drug increased from 9.9% to 17.5% in one decade [8,9]. In fact, *H. pylori* infections refractory to first treatment attempts are becoming more frequent with expanding use of antibiotics for multiple infections and the rising number of patients who undergo *H. pylori* treatment.

After failure of a therapy containing proton pump inhibitor (PPI) and CLARI, either bismuth-based quadruple therapy or PPI–levofloxacin (LVX) triple therapy is recommended by the most recent Maastricht consensus (2012) [2]. Multiple studies and meta-analysis have shown LVX to be a valid alternative to standard antimicrobial agents in first- and second-line regimens [10-12]. It is active even in *H. pylori* strains resistant to CLARI and metronidazole. Eradication rates of patients who harbor these doubly resistant strains are reportedly 92% if the isolates are sensitive to LVX [13]. Levofloxacin-based therapy can be used as second-line treatment in countries with low prevalence of LVX-resistant strains, but should probably be used only as third-line therapy if resistance to this fluoroquinolone is higher than 15% [14].

The *H. pylori* prevalence rate in Portuguese adults is 84.2% [15]. Although the aforementioned empirical triple-therapies are regularly used in the central region of Portugal, to our knowledge, there is no information about the efficacy of these treatments.

This study evaluated eradication rates of a 14-day triple therapy with PPI, amoxicillin and CLARI as empiric first-line therapy and a 10-day triple treatment with PPI, amoxicillin and LVX as a rescue therapy. As secondary aim, we intended to identify clinical and bacterial factors associated with treatment failure.

## Methods

### Patients

In this single-center study, patients with dyspepsia, iron-deficient anemia, need for chronic therapy with PPI and/or first-degree relatives with gastric carcinoma were prospectively considered for inclusion. Each had a recent positive ^13^C-urea breath test (UBT) and indication for upper endoscopy. Exclusion criteria were: age <18 years; pregnancy; lactating and/or fertile women who were not using safe contraceptive methods; history of allergy/hypersensitivity to any antibiotic or PPI; previous gastric malignancy and/or gastric surgery; current use of anticoagulants; marked thrombocytopenia; systemic severe disease (hepatic, cardio-respiratory or renal disease; uncontrolled diabetes; active malignant diseases, coagulopathies); use of antibiotics in the last 4 weeks; use of PPI in the last 2 weeks; intolerance/refusal to undergo upper endoscopy; previous *H. pylori* eradication treatments including fluoroquinolones or known use of this antibiotic for other infections.

They were divided in two groups: Group A—patients who had never received eradication treatments (*n* = 103; 73 female; mean age: 41.6 ± 12.9 years); Group B—patients who failed ≥1 eradication attempts that did not include LVX (*n* = 51; 39 female; mean age: 45.2 ± 13.9 years) and had no documented history of treatment with fluoroquinolones.

### Empiric triple treatments and efficacy assessment

After undergoing upper endoscopies, patients in Group A were immediately prescribed pantoprazole 40 mg 2×/day, amoxicillin 1000 mg 12/12 h and CLARI 500 mg 12/12 h, for 14 days. Patients in Group B were prescribed pantoprazole 40 mg 2×/day, amoxicillin 1000 mg 12/12 h, and LVX 250 mg 12/12 h, for 10 days. We provided detailed explanation of the therapies and potential secondary effects to patients and their families, who were given diaries to record all administrations, side effects and symptoms during therapy. Patients were phoned immediately after treatment to register compliance and any potential symptoms or side effects. They underwent UBT 6–10 weeks after treatment to assess *H. pylori* status. At this time, compliance was confirmed by counting tablets returned by each patient. Treatment was considered complete if all medication was taken. Poor compliance was assumed if <80% of the prescribed drugs were taken.

Patients also returned the diaries they maintained during treatment and were asked to assess tolerability and efficacy of treatment in resolving symptoms by visual scales from 0 to 10 (0: not tolerable at all, 10: excellent tolerability; and 0: not efficacious, 10: fully efficacious, respectively). Adverse events were scored as mild, moderate or severe by their effect on daily activities (daily activities not limited, limited to some extent, or not possible at all, respectively) and need to discontinue treatment. The scoring system was based on that proposed by de Boer et al., with minor modifications [16]. During the study, patients could directly telephone an investigator to resolve any doubts or problems that occurred. Patients were allowed no antibiotics or antisecretory drugs 4 and 2 weeks, respectively, before their UBTs, which were considered positive if *δ* above baseline was ≥4‰.

### Microbiological studies

As this was a research protocol and patients each had indication for upper endoscopy, we took biopsies for *H. pylori* culture, susceptibility testing and genotyping. Minimum inhibitory concentrations (MIC) for amoxicillin, CLARI and LVX, and point mutations in gene *23S rRNA* and in the quinolones resistance-determining region of the *gyrA* gene were determined by a previously published protocol [17]. The *cagA* and *vacA* genotypes were obtained with real-time PCR by using specific primers selected from previously published works [18,19].

### Stopping rules

Efficacy assessment was performed in each group for every 50 included patients. Inclusion was halted if this preliminary analysis revealed eradication rates <80%.

### Statistics

Categorical variables are shown with their relative and absolute values; quantitative variables are expressed as mean ± standard deviation or median + range. The *H. pylori* eradication success was analyzed on both an ITT basis (including all eligible enrolled patients, regardless of compliance with study protocol; patients with unavailable data were assumed to be unsuccessfully treated) and “per protocol” (PP) (including only patients with good compliance and whose data was all evaluable at end of treatment).

Univariate analyses with Student’s *t* test, Mann–Whitney and Fisher’s exact test were used to evaluate associations between treatment effectiveness and age, sex, ethnicity, symptoms/indication for *H. pylori* treatment, body mass index (BMI), location of residence, education level, olive oil, alcohol and tobacco consumption, frequent infections, use of antibiotics in the last 12 months, family history of gastric pathology, *H. pylori* genotype, adverse events during treatment and compliance. Significant variables were subsequently included in a binomial logistic regression that assumed *H. pylori* eradication as the dependent variable. We used the statistical software package SPSS 20.0 (IBM Corp., Armonk, NY, USA).

### Ethics

The study was approved by the ethical committee of our hospital and the Faculty of Medicine, and performed in accordance with the Declaration of Helsinki, the International Conference on Harmonisation of Good Clinical Practice Guidelines, and applicable local laws and regulations. Signed informed consent was obtained from each patient.

## Results

### Demographic variables

During the 4-year period, we included 154 patients (Group A: *n* = 103; Group B: *n* = 51; Table 1). Group B patients received a median of one eradication (range: 1 to 5) before inclusion with the following drugs: amoxicillin: 100%; CLARI: 92.2%; nitroimidazoles: 29.4%. The most common regimen was triple therapy with PPI, amoxicillin and CLARI, followed by PPI, CLARI and nitroimidazoles, and by PPI, amoxicillin and nitroimidazoles.

**Table 1:** **Patients’ demographic and clinical characteristics**

	Group A	Group B
	n = 103	n = 51
Mean age (years)	41.6 ± 12.9	45.2 ± 13.9
	(range 19–77)	(range 18–85)
Sex		
- Male	30 (29.1%)	12 (23.5%)
- Female	73 (70.9%)	39 (76.5%)
Ethnic background		
- European	99 (96.1%)	50 (98%)
- African	4 (3.9%)	1 (2%)
Education level		
- Level 1	30 (29.1%)	19 (37.3%)
- Level 2	73 (70.9%)	32 (62.8%)
Residence		
- Rural	55 (53.4%)	30 (58.8%)
- Urban	48 (46.6%)	21 (31.2%)
Indication(s) for *H. pylori* eradication		
- Non-ulcer dyspepsia	63 (61.2%)	34 (66.7%)
- Iron-deficient anemia	25 (24.3%)	7 (13.7%)
- GERD/Chronic therapy with PPI	24 (23.3%)	17 (33.3%)
- First-degree relatives with gastric cancer	14 (13.6%)	7 (13.7%)
- Peptic ulcer	9 (8.7%)	4 (7.8%)
BMI (Kg/m^2^)	24.6 ± 4.2	25 ± 4
	(range 17.1–37.8)	(range 17.1–33.8)
Alcohol consumption	21 (20.4%)	10 (19.6%)
Smoking	13 (12.6%)	11 (21.6%)
Olive oil consumption ≥1 dl/week	53 (51.5%)	27 (52.9%)
History of frequent infections	20 (19.4%)	13 (25.5%)
Antibiotic consumption in the last 12 months	28 (27.2%)	40 (78.4%)
Family history of gastric diseases	49 (47.6%)	29 (56.9%)

BMI: body mass index; GERD: gastroesophageal reflux disease; PPI: proton pump inhibitors; Level 1: no education or primary school; Level 2: high-school or university grade.

### Compliance, tolerability/adverse events and follow-up losses

Of the 103 patients in Group A, 25 (24.3%) failed to take at least one pill during treatment but only 7 (6.8%) were noncompliant; and 45 Group A patients (43.7%) experienced adverse events (mild: 36; moderate: 8; severe: 1). The patient with severe side effects had an allergic reaction with diffuse rash. Patients’ median assessments of the regimens according to a visual scale, were tolerability: 8 (range: 2–10); efficacy: 8 (range: 0–10). Of the 51 patients in Group B, 3 (5.9%) failed to take at least one pill during treatment but only 2 (3.9%) were noncompliant; and 16 (31.4%) experienced adverse events (mild: 13; moderate: 2; severe: 1). The patient with severe side effects had intractable nausea, vomiting and diarrhea. Patients’ median assessments of treatment according to a visual scale, were tolerability: 8 (range: 2–10) and efficacy: 7 (range :2–9). No patient was lost to follow-up in either group.

### Microbiological studies

Endoscopies with biopsies and culture were successfully and safely performed in all patients. *H. pylori* resistance to CLARI was detected in 22 isolates (21.4%) in Group A, and resistance to LVX in 13 (25.5%) isolates of Group B. Only one case showed resistance to amoxicillin, with a MIC level of 2 mg/L.

Twenty isolates of Group A had point mutations in the *23S rRNA* gene (A2143G/A2142G), and 9 isolates of Group B in the *gyrA* gene. All isolates with mutations had *in vitro* resistance to antibiotics related with those genetic patterns. *H. pylori cagA* and *vacA* genotypes are presented in Table 2.

**Table 2:** ***H. pylori*** **genotypes**

Genotype	Group A	Group B
	(n = 103)	(n = 51)
*cagA* positive	44 (42.7%)	10 (19.6%)
*vacA* s1m1	24 (23.3%)	4 (7.8%)
*vacA* s1m2	28 (27.2%)	12 (23.5%)
*vacA* s2m1	1 (1%)	2 (3.9%)
*vacA* s2m2	50 (48.5%)	33 (64.7%)

### Efficacy of eradication therapy

For Group A, the eradication rate was 68.9% (95% CI 59.4–77.1%) on ITT and 68.8% (95% CI: 58.9–77.2%) on PP analysis. For Group B eradication rate was 52.9% (95% CI: 39.5–66%) on ITT and 55.1% (95% CI: 41.3–68.2%) on PP analysis.

### Factors associated with eradication failure

We successfully treated 85.2% of patients with CLARI-susceptible *H. pylori*, but only 9.1% of patients with CLARI-resistant *H. pylori* (*P* <0.0001). None (0%) of the Group B patients with LVX-resistant infections had successful therapy, compared with 71.1% of patients with LVX-susceptible infections (*P* <0.0001). Antibiotic resistance was independently associated with treatment failure in both groups (Table 3); however multivariate analysis showed only history of frequent infections to independently predict treatment failure in Group A (odds ratio [OR]: 4.24; 95% CI: 1.04–17.24).

**Table 3:** **Risk factors for eradication failure in univariate analysis**

	Triple therapy with clarithromycin			Triple therapy with levofloxacin		
	Failure (n = 32)	Success (n = 71)	*P*/OR (95% CI)	Failure (n = 24)	Success (n = 27)	OR (95% CI);*P*
Female	**27 (84.4%)**	**46 (64.8%)**	**0.043/2.94 (1.01–8.57)**	19 (79.2%)	20 (74.1%)	0.749
Age	41.5 ± 13.7	41.7 ± 12.6	0.967	45.2 ± 10.4	45.3 ± 16.6	0.678
>50 years	9 (28.1%)	22 (31%)	0.820	9 (37.5%)	10 (37%)	1
Caucasian	30 (93.8%)	69 (97.2%)	0.586	23 (95.8%)	27 (100%)	0.471
No education or primary school	20 (62.5%)	53 (74.6%)	0.245	8 (33.3%)	11 (40.7%)	0.772
Urban residence	11 (34.4%)	37 (52.1%)	0.135	12 (50%)	9 (33.3%)	0.265
Non-ulcer dyspepsia	22 (68.8%)	41 (57.7%)	0.383	13 (54.2%)	21 (77.8%)	0.136
GERD/Chronic therapy with PPI	6 (18.8%)	18 (25.4%)	0.616	9 (37.5%)	8 (29.6%)	0.569
First-degree relatives with gastric cancer	5 (15.6%)	9 (12.7%)	0.759	3 (12.5%)	4 (14.8%)	1
Iron-deficient anemia	6 (18.8%)	19 (26.8%)	0.462	4 (16.7%)	3 (11.1%)	0.693
Peptic ulcer	1 (3.1%)	8 (11.3%)	0.268	3 (12.5%)	1 (3.7%)	0.331
BMI	**23.3 ± 3.3**	**25.2 ± 4.4**	**0.035**	26.2 ± 4.4	23.9 ± 3.3	0.064
BMI < 26 kg/m^2^	**29 (90.6%)**	**44 (62%)**	**0.004/5.92 (1.65–21.28)**	13 (54.2%)	21 (77.8%)	0.136
Smoking	7 (21.9%)	6 (8.5%)	0.104	6 (25%)	5 (18.5%)	0.736
Alcohol consumption	4 (12.5%)	17 (23.9%)	0.290	3 (12.5%)	7 (25.9%)	0.300
Olive oil consumption ≥1 dl/week	18 (56.2%)	35 (49.3%)	0.531	11 (45.8%)	16 (59.3%)	0.406
History of frequent infections	**11 (34.4%)**	**9 (12.7%)**	**0.015/3.61 (1.31–9.91)**	7 (29.2%)	6 (22.2%)	0.749
Antibiotic consumption in the last 12 months	12 (37.5%)	16 (22.5%)	0.151	19 (79.2%)	21 (77.8%)	1
Family history of gastric diseases	18 (56.2%)	31 (43.7%)	0.289	11 (45.8%)	18 (66.7%)	0.164
Adverse events	13 (40.6%)	32 (45.1%)	0.830	8 (33.3%)	8 (29.6%)	1
Incomplete treatment	6 (18.8%)	19 (26.8%)	0.462	3 (12.5%)	0 (0%)	0.097
No compliance	2 (6.2%)	5 (7%)	1	2 (8.3%)	0 (0%)	0.216
*CagA* negative	**24 (75%)**	**35 (49.3%)**	**0.018/3.09 (1.22–7.81)**	20 (83.3%)	21 (77.8%)	0.731
*vacA* s1m1	**3 (9.4%)**	**21 (29.6%)**	**0.026/0.25 (0.07–0.90)**	3 (12.5%)	1 (3.7%)	0.331
*vacA* s1m2	7 (21.9%)	21 (29.6%)	0,480	5 (20.8%)	7 (25.9%)	0.749
*vacA* s2m1	0 (0%)	1 (1.4%)	1	0 (0%)	2 (7.4%)	0.492
*vacA* s2m2	**22 (68.8%)**	**28 (39.4%)**	**0.01/3.38 (1.39–8.20)**	16 (66.7%)	17 (63%)	1

BMI: body mass index (kg/m^2^); GERD: gastroesophageal reflux disease.

Significant relationships (i.e., *P* <0.05) are shown in boldface type. Age is shown in years. Odds ratios are shown for significant variables only.

We then repeated the analysis excluding patients with CLARI-resistant strains in Group A and LVX-resistant strains in Group B. In susceptible-only Group A, history of frequent infections (OR: 4.76; 95% CI: 1.24–18.27) and active tobacco consumption (OR: 5.25; 95% CI: 1.22–22.69) were associated with treatment failure. In susceptible-only Group B, noncompliance was higher in patients with treatment failure but not significantly so (18.2% *vs* 0%; *P* = 0.078).

## Discussion

Over the last two decades, widespread use of certain antibiotics has progressively decreased *H. pylori* antimicrobial susceptibility [20-22]. Resistance to CLARI and fluoroquinolones develops rapidly and cannot be overcome by increasing dose or duration of therapy [23]. The only way to identify effective drug combinations is to regularly monitor the success of *H. pylori* eradication and antibiotic resistance in every region.

Although PPI-CLARI-amoxicillin is probably the most common anti-*H. pylori* treatment, its eradication rates have decreased as resistance to antibiotics, particularly macrolides, rises. Almost all consensus statements and reviews still recommend it [2,4,20,24], although some authors suggest longer therapy duration (10–14 days instead of 7 days) or avoiding this regimen in regions where *H. pylori* resistance to CLARI is higher than 15–20% [2,20].

Levofloxacin-based second-line therapies are an encouraging strategy for addressing eradication failures. Multiple studies and meta-analyses show that combined PPI, amoxicillin and LVX regimens have better results, even in patients with more than one failed eradication attempt and CLARI- and metronidazole-resistant strains [10,12,25-27]. Unfortunately, fluoroquinolone resistance is rapidly increasing in many countries [9,28].

The *H. pylori* prevalence rate in Portugal is > 80% [15]. Multiple drugs commonly used for this infection, such as bismuth salts, tetracycline, furazolidone and rifabutin, are not easily available in Portugal, and doxycycline has disappointing results [17]. Although quadruple non-bismuth, sequential and hybrid therapies have been proposed as alternative first-line options, these treatments are complex. Empiric triple-therapy with CLARI is still the preferred first-line treatment and was recommended by the Portuguese Society of Gastroenterology in 2008 [29], with LVX-based treatment recently assuming a major second-line or rescue position. Although by 2000, Cabrita et al. reported *H. pylori* resistance to be 14.6% to CLARI and 11.1% to ciprofloxacin among adults from the Lisbon area [30], and a European multicenter study found resistance rates in the same population of 20.7% for CLARI and 33.3% for metronidazole, the empiric triple treatment remained the most common first-line regimen. In 2013, Megraud et al. reported primary resistance as 31.5% to CLARI and 26.3% to LVX in Portugal [9], and reportedly, obese patients proposed for bariatric surgery in northern Portugal have low eradication rates with CLARI- and LVX-based ITT triple therapies [31]. However, the efficacy of empiric *H. pylori* treatments in Portugal is little studied. To our knowledge, this is the first study of the efficacy of empiric triple regimens among patients in the central region of Portugal.

Our eradication rates were 68.9% for first-line ITT treatment with 14-day PPI-CLARI-amoxicillin and 52.9% for second-line/rescue treatment with 10-day PPI-LVX-amoxicillin. These first-line results are slightly better than the ones published by Cerqueira et al. for patients treated in 2009–2010, and our second-line/rescue results are similar [31].

The main risk factor for treatment failure is undoubtedly antibiotic resistance [7]. We found 21.4% of *H. pylori* isolates were for CLARI-resistant in Group A, and 25.5% were LVX-resistant in Group B. Only two patients with CLARI-resistant *H. pylori*, and none with LVX-resistant *H. pylori,* achieved eradication. Strains of *H. pylori* in Central Portugal are thus often resistant to CLARI and LVX. Notably, although Group B excluded patients with known histories of exposure to fluoroquinolones, its resistance rate to LVX was very high, probably due to common use and misuse of macrolides and fluoroquinolones for respiratory and urinary infections in Portugal and the cross-resistance of *H. pylori* to all macrolides and fluoroquinolones [9,32].

Macrolide-resistant *H. pylori* will certainly become more prevalent in the near future. A recent study of children found a primary resistance rate to CLARI of 34.7% [33]. Clearly macrolides and fluoroquinolones cannot be used to treat *H. pylori* in patients previously exposed to them, as resistance is practically universal in such circumstances [28].

Although we prescribed empiric treatments in patients for whom *H. pylori* susceptibility patterns were determined, this study was a research protocol, the main objective of which was to establish the efficacy of commonly used empiric treatments. Prescriptions were implemented immediately after the endoscopies, but we received antibiotic susceptibility results much later, 2–3 weeks after these procedures. Even among only those patients infected by *H. pylori* strains with no resistance to CLARI in Group A or LVX in Group B, eradication rates were 85.2% and 71.1%, respectively—well below the 90% accepted as the minimum efficacy rate for *H. pylori* treatment regimens [5]. As compliance and adverse event rates did not significantly differ, these factors cannot be considered as responsible for such failures. However, in Group B, treatment failure tended to be higher in noncompliant patients, although not significantly so, due to the limited number of patients.

History of frequent infections was a risk factor for eradication failure in all circumstances, even in patients with no *H. pylori* resistance to CLARI. One possible explanation is that our methodology underestimated *H. pylori* resistance to macrolides. Infection by multiple strains, with different resistance patterns, is common, but subculture testing might mask identification of resistant strains [34,35]. This is a potential cause of treatment failure and negative cultures; thus direct molecular studies of biopsy specimens might be more illuminating [36]. For CLARI, De Francesco et al. recently reported that the prevalence of usually identified point mutations A2143G, A2142G and A2142C might be decreasing [37]. However, we identified mutations in positions A2143G/A2142G of *23S rRNA* gene in 20 of the 22 resistant strains. Notably, the methodology we used does not distinguish A2143G from A2142G, but the clinical significance of this limitation is controversial, although some authors state that the A2143G mutation is the one that significantly lowers *H. pylori* eradication rate [7]. Point mutation A2142C was not detected and we did not search for other mutations; this can be a limitation of our work [37].

Active tobacco consumption was also a risk factor for failure in patients with CLARI-susceptible *H. pylori*. This negative effect is already known and may result from a reduction of antibiotic delivery due to a decreased gastric blood flow, lower intragastric pH or increased activity of the vacuolating toxin activity in gastric cells [2,38].

We had only one case of *H. pylori* resistance to amoxicillin, but that did not determine eradication failure. Resistance to this antibiotic has also been held responsible for treatment failure, but there is no accurate estimate of its effect [39,40].

*H. pylori* genotype can be important as it is reported to be strongly associated with eradication success, antibiotic susceptibility and *H. pylori* virulence factors [14,41,42]. Genotypes *cagA* negative and *vacA* s2m2 induce less inflammation and may contribute to reduced antibiotic delivery and decreased *H. pylori* eradication. Our univariate analysis also suggested this association but logistic regression did not confirm it, although the limited number of patients might have affected these results.

Another possible explanation for our results is that PPI and amoxicillin might have been prescribed in inadequate doses and/or intervals between administrations. Generally, Caucasians metabolize PPI quickly and might need higher doses and frequency of PPI administration [43]. The same might be needed for amoxicillin, as its bactericidal effect depends on % time above MIC and its plasma half-life is very short; thus frequent dosing for amoxicillin is necessary [44]. However, we prescribed the combinations traditionally supported in the literature. Different combinations, with four daily administrations of PPI and amoxicillin, could eventually be tested in future studies.

Various diseases can influence *H. pylori* eradication rates. Patients with peptic ulcers tend to respond better to treatment than patients with non-ulcer dyspepsia [2,45,46]. Although our study found no association between disease manifestations and therapeutic failure, relatively few patients with peptic ulcers were included. The predominance of non-ulcer dyspepsia in our series explains the high number of female patients, as non-ulcer dyspepsia is more common in women [47].

Another limitation of our study was the relatively small study cohort. First, we included only patients with previous positive UBT and indications for upper endoscopy, with very restrictive exclusion criteria, which limited the number of includible patients. Second, when our protocol was designed, no clear rules were in place on interrupting treatment of patients in prospective studies for *H. pylori* treatments. However, Maastricht III consensus established that proposed therapies must achieve eradication rates ≥80%. In following these recommendations, we decided to perform preliminary analyses of each 50 patients and to stop the study if eradication rate was less than 80%. That threshold occurred in the first analysis for Group B and in the second one for Group A. Even stricter stopping rules were more recently proposed by Graham (50 patients; eradication rate <90%) [48].

Finally, some recent studies suggest that 14-day LVX-based triple therapy can provide a >90% *H. pylori* eradication rate, but a 10-day duration may be suboptimal [49]. Although our LVX-treated patients in Group B received a 10-day regimen, 7- and 10-day regimens with LVX were acceptable when our protocol was designed; we chose the longer one specifically to optimize results. Another problem with LVX is lack of consensus about dosage: 250 mg 2×/day, 500 mg 1×/day, or 500 mg 2×/day. The 10-day 500 mg 2×/day dose reportedly has higher eradication rates, but still <80% [50]. Other authors still use a 10-day rescue regimen with PPI, amoxicillin 1 g and LVX 250 mg, all 2×/day [51].

## Conclusions

Empiric triple-therapies with PPI-CLARI-amoxicillin and PPI-LVX-amoxicillin are ineffective as first- and second-line *H. pylori* regimens, respectively, in the central region of Portugal and they are now unacceptable as treatments. The main reason for treatment failure is high *H. pylori* resistance to CLARI and LVX.

History of frequent infections and active tobacco consumption contribute to unsuccessful treatments with CLARI for *H. pylori* strains with no resistance to this drug. In patients with these characteristics, another tailored first-line treatment is preferable.

For first-line empiric treatment, we suggest empiric concomitant or hybrid therapies for 14 days. However, study of the efficacy of these treatments in our region is urgently needed. In light of the scarcity of effective *H. pylori* antimicrobials in our country, if these regimens fail, it is our opinion that second-line treatments must be prescribed according to susceptibility testing.

## Abbreviations

*H. pylori*: *Helicobacter pylori*
ITT: Intention-to-treat
PPI: Proton pump inhibitor(s)
UBT: ^13^C-urea breath test
MIC: Minimum inhibitory concentrations
PP: Per protocol
OR: Odds ratio
